# Dosimetry analysis of panoramic‐imaging devices in different‐sized phantoms

**DOI:** 10.1002/acm2.12059

**Published:** 2017-02-25

**Authors:** Muizz A. Wahid, Ella Choi, David S. MacDonald, Nancy L. Ford

**Affiliations:** ^1^ Oral Biological and Medical Sciences The University of British Columbia Vancouver BC Canada

**Keywords:** paediatric dentistry, panoramic radiography, radiation dosimetry, radiographic phantoms, radiology

## Abstract

The aim of this study is to measure the radiographic dose in adult, adolescent, and child head‐sized PMMA phantoms for three panoramic‐imaging devices: the panoramic mode on two CBCT machines (Carestream 9300 and i‐CAT NG) and the Planmeca ProMax 2D. A SEDENTEXCT dose index adult phantom and custom‐built adolescent and pediatric PMMA dosimetry phantoms were used. Panoramic radiographs were performed using a Planmeca ProMax 2D and the panoramic mode on a Carestream 9300 CBCT and an i‐CAT NG using the protocols used clinically. Point dose measurements were performed at the center, around the periphery and on the surface of each phantom using a thimble ionization chamber. Five repeat measurements were taken at each location. For each machine, single‐factor ANOVA was conducted to determine dose differences between protocols in each phantom, as well as determine the differences in absorbed dose when the same protocol was used for different‐sized phantoms. For any individual phantom, using protocols with lower kVp, mA, or acquisition times resulted in statistically significant dose savings, as expected. When the same protocol was used for different‐sized phantoms, the smaller phantom had a higher radiation dose due to less attenuation of x‐rays by the smaller phantom and differences in the positioning of the ion chamber relative to the focal trough. The panoramic‐mode on the CBCT machines produce images suitable for clinical use with similar dose levels to the stand‐alone panoramic device. Significant dose savings may result by selecting age‐ and size‐ appropriate protocols for pediatric patients, but a wider range of protocols for children and adolescents may be beneficial.

## 1 Introduction

Panoramic radiography is a two‐dimensional x‐ray examination that produces an image of the dental arches.^1^ Due to advances in dental cone beam computed tomography (CBCT), manufacturers are including additional functionality including panoramic‐imaging capabilities to provide an all‐in‐one system for dental imaging. In some cases, the additional functionality includes a reconstructed panoramic image obtained from a CBCT dataset and reformatting within the software, which may remove some of the superposition of structures that are expected in the panoramic image. Software reformatting provides a series of useful images from a single acquisition (3D volumes, 2D slices through anatomy of interest, 2D panoramic view, etc.) but clinicians must remember that the dose for the 3D acquisition is higher than for panoramic imaging^2^ and this technique should only be done if the 3D images are required. However, some CBCT machines also offer a panoramic acquisition to obtain a true 2D image that is advertised as comparable with stand‐alone machines, with some manufacturers’ boasting a reduction in dose over stand‐alone panoramic‐imaging systems. To date, no dosimetry studies have been reported in the scientific literature to compare the dose received by the patient or the dose distribution in combination units compared with single‐function panoramic‐imaging machines.

Panoramic machines often use pre‐set imaging protocols with various exposure parameters (kVp, mA, acquisition time) that determine the radiation dose to a patient. Helmrot et al. have suggested using the dose area product (DAP) as a standardized dose metric for all dental radiography^3^ due to the convenience of measurements and that the DAP is measured independent of the patient and can therefore be specified by the manufacturer. The DAP has been used to establish radiation output reference levels in Greece^4^ and Germany,^5^ although the reference levels are highly dependent on the measurements used to find the 75% dose level, but are not for defining absorbed or effective doses. Although the DAP values are obtained at the tube port in an empty field, and therefore not including the beam dispersion or scattering effects within the patient, they have been used to estimate the effective doses for patients using published conversion factors.^3^, ^5^ However, the results are highly variable depending on the measurement techniques and whether the salivary glands were included in the effective dose calculations;^5^ the tissue‐weighting factors were updated in ICRP 2007, which assigned a weighting factor to the salivary glands instead of including them in the remainder tissues.^6^ Roberts et al. have also shown that effective doses calculated using older tissue‐weighting factors (from 1990) for dental imaging are roughly half that using the factors published in 2007, six primarily due to the inclusion of the salivary glands in the calculation,^7^ which limits the applicability of effective dose measurements. We propose using the absorbed dose measurement within a head‐sized PMMA phantom, as it represents the energy absorbed within the phantoms, including dose from both the primary and scattered radiation, and can be used to estimate other metrics if desired.

Although dental imaging contributes less than 0.1% of the radiation dose the global population receives, radiation risk should always be considered when conducting panoramic radiography.^2^ The radiation risk is three times greater in patients that are less than 10 yr old compared to those that are above 30 yr.^8^ The increased radiosensitivity of tissues in children, along with their longer anticipated life span post‐exposure, increases their risk of developing cancer over their lifetime.^9^ The radiosensitive nature of pediatric patients validates the need to carefully monitor the radiation exposure to these patients in particular. There are very few studies examining the radiographic dose on pediatric patients from panoramic radiography, and none when using the panoramic‐mode on a CBCT unit. A study conducted by Hayakawa et al. examined the doses in a dry‐skull phantom representing a 5–6 yr old child for two single‐function panoramic machines.^10^ Comparing adult and child imaging protocols for the phantom, Hayakawa et al. concluded that pediatric exposure settings reduce dose irrespective of machine. Choi et al. have developed two pediatric head‐sized PMMA phantoms, representing a child aged 5 yr and an adolescent aged 12 yr, and measured the absorbed dose in various locations in dental CBCT,^11^ leading to the same conclusions that pediatric exposure settings could dramatically decrease patient doses.

The aim of this study was to measure and compare the absorbed dose in adult, adolescent, and child head‐sized PMMA phantoms for three panoramic‐imaging devices: the panoramic modes on the Carestream 9300 CBCT and i‐CAT Next Generation CBCT, and the Planmeca ProMax 2D panoramic machine. The study also aims to establish the importance of selecting patient‐appropriate protocols, particularly in pediatric patients.

## 2 Methods and materials

### 2.A Phantoms

**Figure 1:**
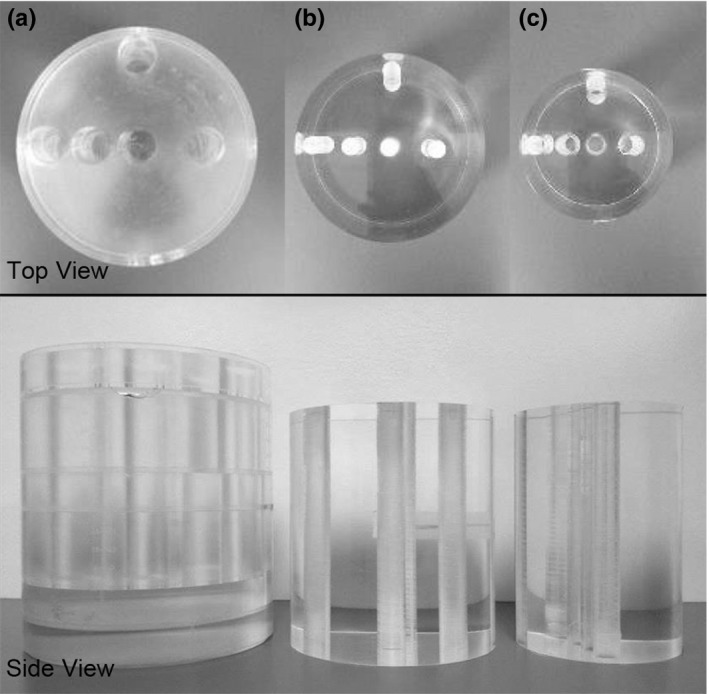
Comparison of different head‐sized PMMA phantoms from top and side view. (a) Adult 160 mm diameter × 162 mm height, (b) Adolescent 135 mm diameter × 150 mm height, (c) Child 100 mm diameter × 150 mm height.

Cylindrical polymethyl methacrylate (PMMA) phantoms containing five holes drilled through the height of the cylinder for ionization chamber placement (Fig. 1) were used to measure the dose. The adult phantom was the commercially available SEDENTEXCT DI (Leeds Test Objects, Ltd, York, UK) measuring 160 mm diameter × 162 mm height. The adolescent and pediatric phantoms were designed in our lab and custom‐built (British Columbia Cancer Agency, Genome Sciences Center, Vancouver, Canada). The adolescent phantom (135 mm diameter × 150 mm height) was designed to represent a 12‐year‐old child beginning orthodontic treatment, whereas the child phantom (100 mm diameter × 150 mm height) was designed to represent a 5‐year‐old child.^11^ The dimensions of the custom‐built phantoms were obtained from measuring anatomic reference points in the dental CBCT images of pediatric patients.

A panoramic radiograph of a Pan DXTTR (Rinn Corporation, Elgin IL, USA) was taken by each machine to assess their images. The DXTTR phantom is an anthropomorphic phantom comprised of a natural bone skull embedded in resin. The head has detailed facial features to enable positioning using anatomical landmarks and is mounted on a tripod that can articulate to enable angling the head to align with the laser positioning guides.

### 2.B Imaging systems

Panoramic radiographs were performed using the panoramic‐mode on two CBCT machines, the Carestream 9300 (Carestream, Rochester NY, USA) and i‐CAT NG (Imaging Sciences International, Hatfield PA, USA), and using a stand‐alone panoramic machine, the ProMax 2D (Planmeca Helsinki, Finland). Although panoramic images can be obtained by reformatting a 3D CBCT image, both machines used in this study have a separate panoramic‐imaging acquisition mode; it is the panoramic‐imaging acquisition mode that was used in this study. All of the devices studied were equipped with digital detectors, a TFT detector for the Carestream 9300, a‐Si flat panel detector with a CsI scintillator for the i‐CAT NG and a CCD detector for the Planmeca Promax 2D.

A PMMA plate was mounted onto a tripod upon which the PMMA phantoms were situated. Positioning lasers were used to place each PMMA phantom within the field of view (FOV). Specifically, the front peripheral hole within the phantom was positioned in the imaging focal trough and the phantom was centered vertically within the field of view. Phantoms were exposed to acquisition protocols deemed clinically appropriate with regard to phantom dimensions. All protocols used in the study are pre‐set within their respective machines except the small child protocol (ProMax 2D) for which the exposure parameters (kVp, mA, and acquisition time) were manually adjusted to match the posted clinically accepted technique chart. Table 1 shows the exposure parameters used for each protocol for the panoramic‐mode on the CBCT machines and the stand‐alone machine, respectively.

**Table 1:** Comparison of panoramic‐imaging protocols for Carestream 9300 and i‐CAT NG combination machines with the Planmeca ProMax 2D stand‐alone machine. Image sizes (width x height) are included for the average adult setting

Protocol	Time (s)	kVp	Tube current (mA)	DAP (mGycm^2^)	Phantoms
Carestream 9300 (260 × 149 mm)					
Child	13.2	64	10	58.6	Adolescent, child
Small adult	13.6	68	8	72.4	Adult, adolescent
Average adult	14.3	70	10	104	Adult, DXXTR
Large adult	15.3	74	10	130	Adult
i‐CAT NG (316 × 153 mm)					
Small	18.3	84	5	91	Adolescent, child
Large	20	94	5	146.4	Adult, adolescent, DXXTR
Planmeca promax 2D (233 × 114 mm)					
Small child (<7 yr)	14	60	4	29	Child
Child (7–12 yr)	14	62	5	39	Adolescent, Child
Adolescent	16	64	7	76	Adolescent
Small adult	16	66	9	103	Adult, adolescent
Average adult	16	68	13	158	Adult, DXXTR
Large adult	16	70	14	180	Adult

**Figure 2:**
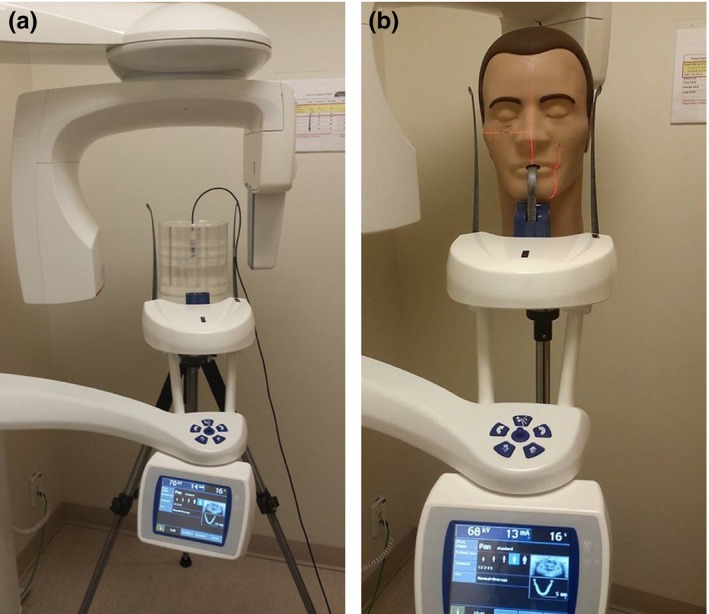
Adult‐sized PMMA phantom and DXTTR phantom positioned in the Planmeca ProMax 2D. (a) PMMA set‐up. (b) DXTTR set‐up.

The Pan DXTTR phantom was situated within each device using positioning lasers to ensure that the mandible of the phantom was in the focal trough. Panoramic radiographs were performed using the average adult protocol in the Carestream 9300 and ProMax 2D, and the large protocol in the i‐CAT NG. The set‐up of the adult PMMA and DXTTR phantoms are shown for the Planmeca ProMax 2D machine (Fig. 2). Pan DXTTR images were viewed by a maxillofacial radiologist to determine if the CBCT machines were producing images that were consistent with a direct panoramic‐imaging acquisition (including the superposition of overlying structures), and to verify that the images were of clinical use, and could potentially replace the stand‐alone panoramic device.

### 2.C Dosimetry measurements

A thimble ion chamber (10 × 6‐0.6‐CT; Radcal Corporation, Monrovia, CA), with an active volume of 0.6 cm^3^, was placed in either the center or peripheral hole, ensuring that the midline of the chamber was centered vertically in the phantom. Holes that were not utilized by the ion chamber were filled with cylindrical PMMA plugs; thereby, no air pockets were present within the phantom. For the surface measurements, the ion chamber was taped to the outer surface of the phantom, ensuring that the midline of the chamber was again centered vertically in the phantom. An Accu‐Dose meter (model 2186 v. 7.03; Radcal Corporation) measured the absorbed dose. The ion chamber was calibrated at the factory to be within ±5% over the range of energies used in diagnostic CT. Utilizing the high‐sensitivity setting improved the accuracy of the measurements. Five measurements for the absorbed dose were taken at each measurement location; measurements were obtained in the center of the phantom, and in four locations (front, back, left and right) around the periphery and the same four locations on the surface of the phantom for each protocol to provide nine measurement locations for each phantom.

### 2.D Data analysis

The mean and standard deviations of the five repeated dose measurements are reported in μGy and dose measurements normalized by the tube current in milliamps and exposure time are given as μGy/mAs. All statistical analysis was done using GraphPad Prism^®^ (version 6.0 h, GraphPad Software, San Diego California, USA). For each phantom, a single‐factor ANOVA (*α* = 0.05) and Sidak's multiple comparisons test was conducted using the absorbed dose values to determine statistically significant differences between protocols for each machine. An additional single‐factor ANOVA (*α* = 0.05) and Sidak's multiple comparisons test was conducted using the normalized absorbed dose value (μGy/mAs) to determine statistically significant differences between the Carestream 9300, i‐CAT and ProMax 2D for equivalent protocols used for each phantom.

## 3 Results

The absorbed dose in adult‐, adolescent‐, and child‐sized phantoms was measured for the panoramic‐mode of the Carestream 9300 (Table 2), the panoramic‐mode of the i‐CAT NG (Table 3) and the Planmeca ProMax 2D (Table 4). Each table shows the measurements obtained in a single device for all phantoms, comparing the different imaging protocols available on each imaging device. Using different imaging protocols within a single phantom resulted in statistically significant changes in radiation dose at every location for each machine (*P*<0.01), as expected. For each imaging device, when the same protocol was used for different‐sized phantoms, the smaller phantom generally had a higher dose measured at every location within the phantom for each imaging modality (*P*<0.0001).

**Table 2:** Carestream 9300 measurements: Average absorbed dose value given in μGy. Standard deviation is denoted by ±

Radial distance	Chamber location	Adult phantom (μGy)	Adolescent phantom (μGy)	Child phantom (μGy)
Child Protocol				
Center			920 ± 2	1355 ± 18
Periphery	Front		116 ± 2	205 ± 1
	Left		232 ± 3	251 ± 4
	Right		177 ± 4	203 ± 1
	Back		615 ± 3	1090 ± 6
Surface	Front		50 ± 2	95 ± 3
	Left		119 ± 2	145 ± 2
	Right		94 ± 3	100 ± 1
	Back		463 ± 2	708 ± 2
Small adult protocol				
Center		639 ± 3	943 ± 6	
Periphery	Front	92 ± 2	124 ± 5	
	Left	290 ± 4	221 ± 2	
	Right	481 ± 11	248 ± 4	
	Back	499 ± 2	626 ± 3	
Surface	Front	34 ± 2	57 ± 3	
	Left	388 ± 5	111 ± 2	
	Right	473 ± 4	224 ± 3	
	Back	394 ± 4	479 ± 1	
Average adult protocol				
Center		926 ± 6		
Periphery	Front	146 ± 1		
	Left	528 ± 5		
	Right	1317 ± 5		
	Back	708 ± 5		
Surface	Front	52 ± 2		
	Left	774 ± 2		
	Right	826 ± 1		
	Back	572 ± 3		
Large adult protocol				
Center		1176 ± 8		
Periphery	Front	177 ± 1		
	Left	1318 ± 12		
	Right	1571 ± 3		
	Back	857 ± 6		
Surface	Front	69 ± 2		
	Left	936 ± 4		
	Right	1007 ± 2		
	Back	668 ± 2		

**Table 3:** i‐CAT NG measurements: Average absorbed dose value given in μGy. Standard deviation is denoted by ±

Radial distance	Chamber location	Adult phantom (μGy)	Adolescent phantom (μGy)	Child phantom (μGy)
Small protocol				
Center			454 ± 4	768 ± 4
Periphery	Front		162 ± 1	262 ± 6
	Left		623 ± 3	447 ± 4
	Right		637 ± 6	1139 ± 5
	Back		263 ± 5	379 ± 1
Surface	Front		68 ± 3	111 ± 3
	Left		496 ± 2	328 ± 2
	Right		429 ± 3	888 ± 4
	Back		194 ± 1	265 ± 3
Large protocol				
Center		653 ± 5	760 ± 8	
Periphery	Front	187 ± 3	267 ± 4	
	Left	680 ± 3	781 ± 4	
	Right	666 ± 3	794 ± 6	
	Back	363 ± 4	400 ± 2	
Surface	Front	72 ± 4	116 ± 3	
	Left	476 ± 2	603 ± 3	
	Right	463 ± 8	559 ± 6	
	Back	263 ± 4	298 ± 5	

**Table 4:** Planmeca ProMax 2D measurements: Average absorbed dose value given in μGy. Standard deviation is denoted by ±

Radial distance	Chamber location	Adult phantom (μGy)	Adolescent phantom (μGy)	Child phantom (μGy)
Small child protocol				
Center				580 ± 14
Periphery	Front			110 ± 2
	Left			134 ± 2
	Right			155 ± 1
	Back			426 ± 5
Surface	Front			41 ± 3
	Left			44 ± 3
	Right			96 ± 2
	Back			303 ± 4
Child protocol				
Center			493 ± 8	780 ± 3
Periphery	Front		65 ± 1	154 ± 3
	Left		220 ± 1	184 ± 2
	Right		174 ± 2	214 ± 2
	Back		388 ± 3	580 ± 2
Surface	Front		22 ± 3	59 ± 3
	Left		136 ± 5	60 ± 2
	Right		83 ± 1	136 ± 2
	Back		321 ± 3	413 ± 3
Adolescent protocol				
Center			808 ± 9	
Periphery	Front		112 ± 2	
	Left		402 ± 7	
	Right		247 ± 4	
	Back		638 ± 2	
Surface	Front		44 ± 4	
	Left		250 ± 4	
	Right		173 ± 6	
	Back		499 ± 5	
Small adult protocol				
Center		695 ± 6	1127 ± 6	
Periphery	Front	222 ± 3	156 ± 4	
	Left	1187 ± 9	625 ± 1	
	Right	1062 ± 2	441 ± 3	
	Back	555 ± 5	891 ± 2	
Surface	Front	67 ± 5	66 ± 7	
	Left	1284 ± 9	358 ± 4	
	Right	1052 ± 6	185 ± 6	
	Back	438 ± 3	697 ± 2	
Average adult protocol				
Center		1102 ± 3		
Periphery	Front	352 ± 5		
	Left	1935 ± 17		
	Right	1637 ± 9		
	Back	864 ± 6		
Surface	Front	109 ± 3		
	Left	2001 ± 11		
	Right	1603 ± 6		
	Back	683 ± 2		
Large adult protocol				
Center		1268 ± 4		
Periphery	Front	391 ± 4		
	Left	2061 ± 9		
	Right	1935 ± 7		
	Back	988 ± 1		
Surface	Front	128 ± 2		
	Left	2294 ± 13		
	Right	1867 ± 12		
	Back	777 ± 2		

**Figure 3:**
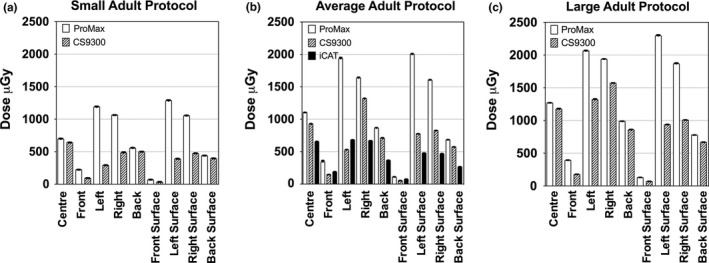
Absorbed doses measured in the adult PMMA phantom using protocols designed for (a) small adults, (b) average adults, and (c) large adults. Since the i‐CAT only has one adult protocol, the measurements are only included in the average adult plot (b). All measurements are significantly different except as noted (*P*<0.001).

**Figure 4:**
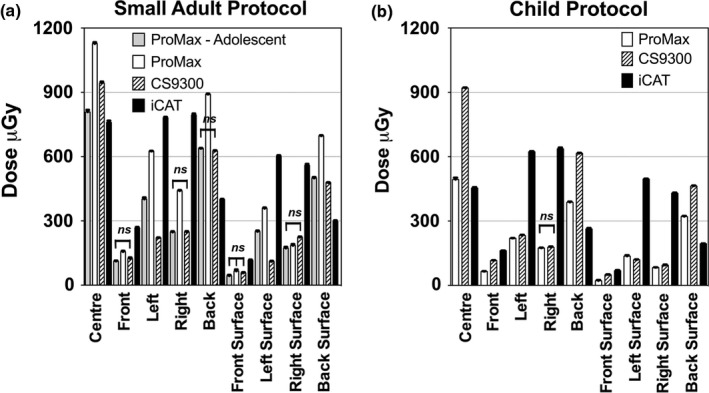
Absorbed doses measured in the adolescent PMMA phantom using protocols designed for (a) small adults, and (b) children. Since the Promax 2D was the only machine with an adolescent protocol, the measurements were included with the small adult plot (a). All measurements are significantly different except as noted (*P*<0.001).

**Figure 5:**
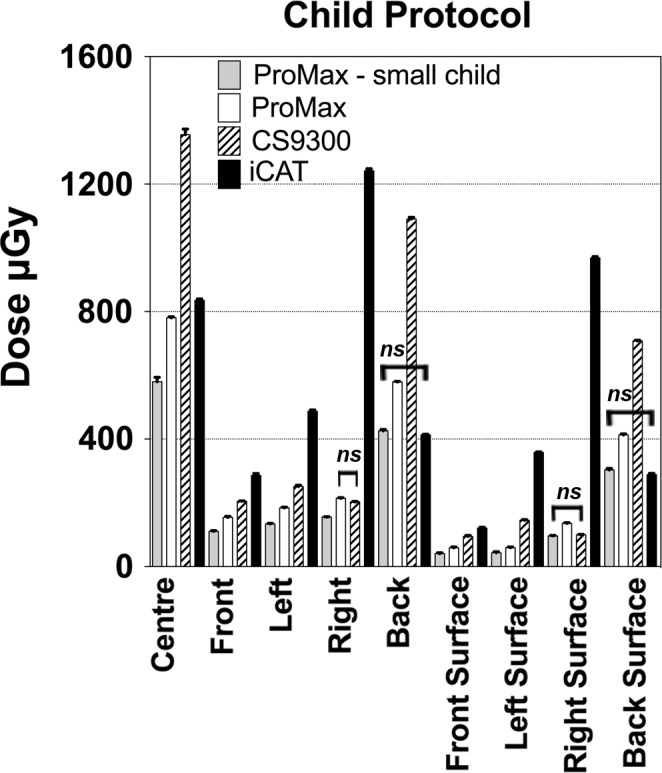
Absorbed doses measured in the child PMMA phantom using protocols designed for children. All measurements are significantly different except as noted (*P*<0.001).

The same imaging protocol was also compared for each phantom using matched imaging protocols across the different machines for the adult phantom, adolescent phantom and child phantom. To compare the different machines, absorbed doses measured in each location within and on the surface of the phantom are plotted for the adult phantom in Fig. 3, the adolescent phantom in Fig. 4 and the child phantom in Fig. 5. All absorbed doses were statistically significant (*P*<0.001) except as indicated on the graphs. The adolescent setting on the ProMax 2D system seemed similar to the small adult setting on the CS 9300 machine, with a few measurements in the peripheral regions showing no difference between the machines (Fig. 4).

**Figure 6:**
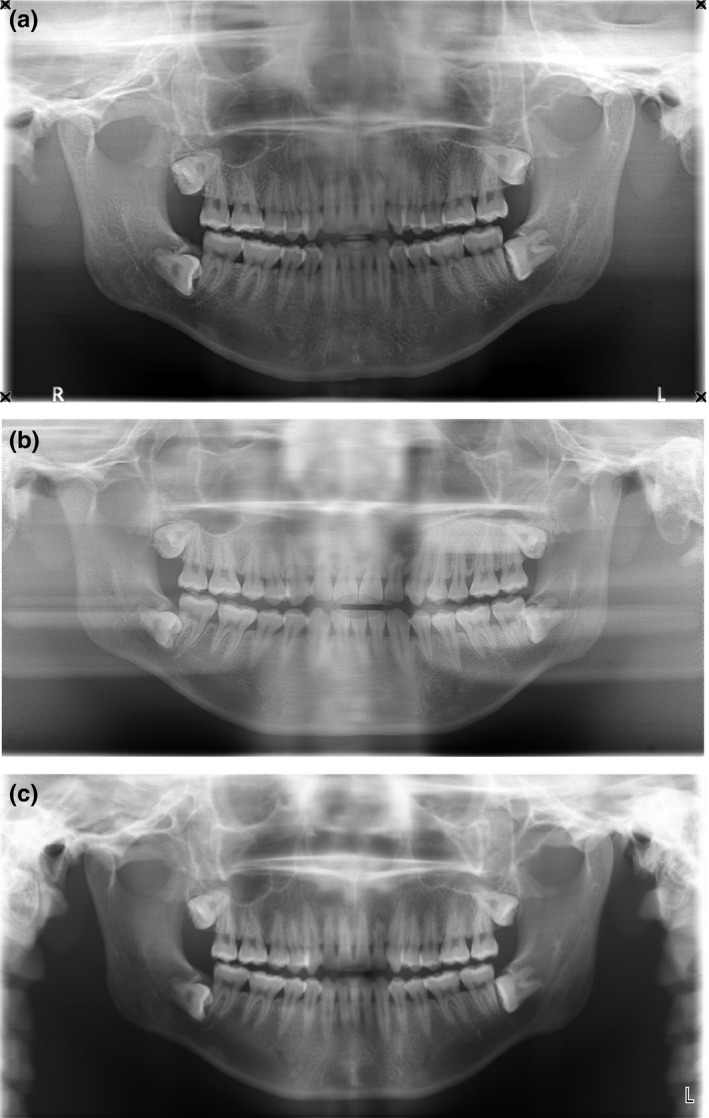
Panoramic images of the DXXTR phantom using the average adult settings on the (a) CS 9300, (b) i‐CAT NG, and (c) ProMax 2D. Images (a) and (b) are produced by units which are primarily CBCT, whereas (c) is produced by a dedicated panoramic radiographic unit. It is clear that the almost wholly dentate human skull (which includes all four impacted third molar teeth) imbedded in this DXXTR phantom has a missing lower incisor. Although (c) displays the full length of all teeth including the incisors, indicating that they (and those of (a)) are within the anterior focal trough for these units, whereas those in (b) display only the crowns, but not the apices which are outside the anterior focal trough. (b) displays more obviously the superimposition of the vertebral column and secondary images of the contralateral mandible. These differences reflect the standard positioning of the phantom as a patient in each of the three units during the exposure.

Panoramic radiographs of the Pan DXTTR phantom using the average adult settings on the Promax 2D and Carestream 9300, and the large setting on the i‐CAT NG are shown in Fig. 6. The images from the Promax 2D (Fig 6c) and the CS9300 (Fig. 6a) both shows the full length of the teeth, which are in the focal trough. However, the i‐CAT image (Fig. 6b) does not show the apices, which are outside of the focal trough in this image. In all three images, the vertebral column is visible, and superposition of structures is evident, including the ghost images of the contralateral mandible. The images obtained from the 2 CBCT combination units are clearly obtained with a true panoramic 2D acquisition and were deemed to be of clinical use similar to the images produced by the stand‐alone unit by a maxillofacial radiologist.

## 4 Discussion

### 4.A Dose at different locations within a phantom

The dose distribution between the devices contain enough similarities to indicate that the panoramic‐mode on the Carestream 9300 and i‐CAT NG CBCT machines use a similar trajectory as the ProMax 2D when obtaining a panoramic image; for panoramic machines, the center of rotation changes during the acquisition, as opposed to a CBCT acquisition, which has a fixed center of rotation. We believe that the dose distribution indicates that the center of rotation changes throughout the scan when the CBCT machines operate in the panoramic acquisition mode. Clinicians must be also aware that the rotation of the x‐ray tube behind the patient results in a non‐uniform radiation dose distribution within a patient. The radiation doses were the lowest at the front of the phantom for peripheral and surface measurements in each machine due to the trajectory of the x‐ray tube as expected. In the adult phantom, the highest doses were generally measured at the lateral peripheries for all devices. In larger phantoms, the sides are closer to the x‐ray tube during its rotation increasing the radiation per unit area. The temple support is also in closer proximity to the sides of the adult phantom, facilitating radiation scatter at those peripheries. The highest dose was measured at the center in both adolescent and child phantoms for the Carestream 9300 and ProMax 2D. X‐rays pass through the center of these smaller diameter phantoms throughout the x‐ray tube's trajectory, increasing the measured dose. X‐rays must also travel a greater distance before reaching the adolescent and child phantoms relative to the adult phantom, increasing beam divergence from the x‐ray tube. Consequently, a lower radiation per unit area may result at the peripheries in smaller diameter phantoms. For the i‐CAT NG, the lateral peripheries generally had the highest dose in both adolescent and child phantoms. The proximity of a rotation center to a side of the phantom affects dose measurements within a phantom, and the distance can differ between manufacturers’ equipment.^12^ As a result, manufacturing differences (i.e., rotation center, beam geometry) between the Carestream 9300, i‐CAT NG and the ProMax 2D may contribute to the observed differences in relative doses at each location. Surface dose measurements were generally the lowest due to minimal backscatter radiation.

Deman et al. measured the radiographic dose at various locations in a SEDENTEXCT DI adult phantom for the ProMax 2D using the large adult protocol.^2^ The study reported the highest dose at the center of the phantom (6.66 μGy/mAs), followed by the back periphery (5.86 μGy/mAs), and then the left and right peripheries (2.77 μGy/mAs and 2.15 μGy/mAs, respectively). In agreement with this study, the lowest dose was measured at the front periphery (0.76 μGy/mAs) due to the trajectory of the x‐ray tube. Differences in absorbed dose and dose distribution between Deman et al. and the data shown here may be attributed to the positioning of the phantom within the machine. Explicit instruction on the placement of the phantom was not reported by Deman et al. whereas this study ensured that the front peripheral hole was located in the focal trough for all phantoms, as determined using the positioning lasers to align the phantom within the focal trough of the machine.

### 4.B Comparing different protocols within the same phantom

Increasing exposure parameters (kVp, mA, and acquisition time) resulted in significant dose increases, whereas decreasing exposure parameters resulted in significant dose savings at all locations within a phantom, as expected. The one exception was the measurements taken on the left side of the adolescent phantom imaged using the child protocol and the small adult protocol on the CS9300; in this case, the small adult protocol showed slightly lower doses which could be due to a slight difference in positioning the phantom within the focal trough of the imaging system or could mean that the dose levels are similar in that location between the two protocols. Imaging protocols pre‐set within each device must be chosen appropriately for each patient based on size and age. Although protocols with lower exposure parameters result in significant dose savings, the diagnostic quality of images should not be compromised. Studies have observed image quality deterioration as a result of a reduction in exposure parameters.^13^

### 4.C Comparing the same protocols with different phantoms

For identical imaging parameters, the smaller phantom generally has a higher radiation dose. A couple of exceptions were observed for the left and/or right sides of the phantoms; in a few cases, the smaller phantom showed higher doses under the same imaging conditions (adolescent vs. adult on the CS9300 small adult protocol and the Promax 2D small adult protocol, child vs. adolescent for the i‐CAT small protocol and Promax 2D child protocol). One possible explanation is the smaller phantoms had their measurements in different locations within the x‐ray beam due to the curvature of the phantom and fixed hole positions, which positioned the ion chamber in a different location relative to the machine's focal trough. The smaller diameter allows x‐rays to directly reach more areas on the phantom during any point of the x‐ray tube's trajectory. Less attenuation of x‐rays through the PMMA also increases the radiation dose measured at the center and front of the smaller phantoms. Clearly, children may be exposed to unnecessary radiation dose if exposure parameters are not adjusted appropriately. Minimizing radiation exposure to children is of primary concern due to the increased radiosensitivity of their tissues. Children also have a longer life span, increasing the risk of cancer induction during their lifetime.^14^

### 4.D Comparing pediatric protocols between the devices

Differences in kVp, mA, and acquisition time result in differences in absorbed dose between the devices. In order to compare the effect of different imaging modalities on dose within a phantom, dose measurements were normalized for mAs. The i‐CAT NG generally had the highest normalized doses within the child phantom, and the Carestream 9300 generally had the lowest normalized doses. Consequently, manufacturing differences (location of rotation center, beam geometry) between the Carestream 9300, i‐CAT, and ProMax 2D may also contribute to the differences in doses at each location. For example, the kVp for each protocol within the Carestream 9300 changes throughout an exposure potentially causing differences in dose distribution and absolute dose within the phantom relative to the other machines. Image quality is invariably dependent on equipment; however, the CBCT machines produce panoramic images that are of comparable diagnostic quality to the ProMax 2D (Fig. 6).

The pediatric protocols available within the Carestream 9300, i‐CAT NG, and ProMax 2D decrease the radiation exposure within children. However, the devices used in the study only had one pre‐set child protocol available, and only the ProMax 2D had an adolescent setting. For the ProMax 2D, manually inputted exposure parameters based on the University of British Columbia Dentistry guidelines for panoramic radiographs were used to expand the imaging protocols available for children. The importance of providing a spectrum of pediatric (and adolescent) imaging protocols prevents clinicians from over‐ and under‐estimating the radiation exposure received by the variety of children seen in a dental clinic. From our dosimetric comparisons, it is clear that the stand‐alone panoramic‐imaging system had a clear advantage over the combination units, because we had the flexibility to manually adjust the imaging parameters for smaller children, and there were more pre‐set protocols for the pediatric population available.

The use of PMMA phantoms in this study allows for measurements of the absorbed dose; however, measurements within a uniform phantom do not accurately represent the dose distribution within a patient. To more accurately reflect patient dose, phantoms with bony anatomy representing each of the age demographics (adult, adolescent, and child) should be developed for future studies. The bony structures will alter the absorption and scatter properties of the phantom, giving a more realistic dose distribution. Furthermore, having tissue equivalent material will also enable estimates of the radiation risk using ICRP weighting factors.

## 5 Conclusion

The panoramic‐mode on the CBCT machines studied produced diagnostic quality images with comparable radiographic dose to a stand‐alone panoramic‐imaging device. Smaller phantoms receive more radiation when imaging protocols are identical for each device. The study demonstrates that pediatric protocols reduce the radiographic dose to children, but the combination units had a limited number of protocols available. All panoramic‐imaging devices, both stand‐alone and combination units, will benefit from including a larger range of pre‐set options representing the pediatric and adolescent populations.

## Conflict of interest

The authors have no conflicts of interest to disclose.
